# The Impact of Radiographic, Metabolic and Demographic Characteristics on Kidney Stone Recurrence

**DOI:** 10.3390/jpm12101632

**Published:** 2022-10-02

**Authors:** Igal Shpunt, Hadar Pratt Aloni, Nelli Khanukaeva, Pearl Herskovitz, Ishai Dror, Brian Berkowitz, Dan Leibovici, Yaniv Shilo

**Affiliations:** 1 Department of Urology, Kaplan Medical Center, Affiliated with the Hebrew University, Rehovot 7661041, Israel; 2 Department of Imaging, Kaplan Medical Center, Affiliated with the Hebrew University, Rehovot 7661041, Israel; 3 Department of Earth and Planetary Sciences, Weizmann Institute of Science, Rehovot 7610001, Israel

**Keywords:** urolithiasis, renal colic, clinical score, follow-up

## Abstract

Urolithiasis is a frequent disease with cited rates of recurrence after initial diagnosis that vary widely and range between 35% and 50%. We assessed the radiographic recurrence rate in patients with urinary stones and its risk factors. We retrospectively identified patients who were diagnosed with urinary stones on non-contrast computed tomography from 2010 to 2011, and underwent another imaging examination at least six months afterwards. We collected patient demographic, clinical, laboratory and radiologic data and compared patients with and without urinary stone recurrence. Ultimately, 237 patients were included in the study; the mean follow-up was 6.7 years; 88 patients (37.1%) had recurrence based on our recurrence criteria. On univariate analysis, the significant parameters for recurrence were baseline serum calcium and uric acid, stone location in the kidney, surgical intervention and stone burden volume. On multivariate analysis, surgical intervention (OR 3.07, *p* = 0.001), baseline calcium (OR 2.56, *p* = 0.011), baseline uric acid (OR 1.30, *p* = 0.021) and stone location in the kidney (OR 2.16, *p* = 0.012) were associated with higher risk of recurrence. These findings may guide personalized follow-up protocols for patients with urolithiasis based on their risk factors.

## 1. Introduction

Nephrolithiasis is a common disease, with prevalence ranging from 5–9% in Europe to as high as 7–13% in the United States, with rising incidence in the past few decades [1].

The overall recurrence rate of renal stones reported in the literature varies highly and relies mainly on a few studies from the 1970s–1990s with relatively small sample sizes (e.g., less than 200 patients). Only a small number of studies since 2000 were conducted on this subject, albeit with somewhat larger sample sizes. Moreover, there is considerable inconsistency in the definition of “recurrence” among studies. While some authors consider recurrence as a clinical manifestation, i.e., stone passage or symptoms [2,3], others rely solely on radiologic findings [4]. Older studies frequently cited for recurrence rates are based on self-reporting of stone-related symptoms in questionnaires [5,6]. Different risk factors for stone recurrence have been reported such as male gender, stone composition, and metabolic syndrome (MetS), yet controversies in the medical literature exist regarding whether these risk factors are indeed contributors for stone recurrence.

Our goal is to broaden the knowledge of this important issue by: (1) assessing the rate of stone recurrence based on a clear definition of “recurrence”, namely, only new stones detected per imaging session (our defined criteria for stone recurrence are detailed in the Materials and Methods section); (2) determining the impact of different radiologic findings on recurrence rate including characteristics and burden of stones and findings linked to MetS, and (3) reassessing demographic and laboratory findings, such as those found in previous studies (e.g., age, serum uric acid), all as predictors for recurrence rate. 

## 2. Materials and Methods

The institutional review board approved this study. This is a cohort study, based on medical records of patients diagnosed with kidney and/or ureteral stones per computed tomography (CT) scan performed at our center between May 2010 and March 2011, to allow long-term follow-up. The study is retrospective, anonymous, and without active intervention on patients. 

### 2.1. Study Population

Patients eligible for the study were adults >18 years old, with at least one kidney or ureteral stone >3 mm found on a non-contrast computed tomography (NCCT) scan performed at our hospital between May 2010 to March 2011. Additionally, patients had a successive urinary imaging: NCCT, contrast enhanced computed tomography (CCT), ultrasound (US) or kidney-ureter-bladder x-ray (KUB) at least 6 months after the first NCCT.

### 2.2. Study Outcomes and Variables

*Dependent variables:* The main outcome of the study is radiographic recurrence, defined, per imaging, as one of the following: (1) new kidney or ureteral stone on contralateral side, or (2) new stone on ipsilateral side but in a different location.

Stones that moved distally from their initial location on the first NCCT were not defined as recurrence. If there were multiple imaging scans, the first identification of a new stone was chosen. Patients who underwent a surgical procedure to remove a stone were reassessed at the end of the procedure by a review of a new imaging examination to clarify the post-operative stone status. Once a patient had a recurrence, follow-up ended.

*Independent variables:* Epidemiological data were collected from patient medical records, and radiologic information was gathered from the institutional imaging software (Algotec PACS, version 11, Minnetonka, MN, USA).

(1) Demographic data: Gender, age at first CT with stones.
(2) Baseline CT stone data:
  (a) Stone burden: Several stone burden measurements tools are available which correlate to each other [7]. Here, we employed the widely accepted ellipsoid formula of the European Association of Urology [8]: Stone volume = length × width × depth × (π/6). Dimensions were measured on three axes. In cases of multiple stones, a calculation was performed for each stone separately and addition was performed afterwards. Units are in mm^3^.
  (b) Stone density: Measured in Hounsfield units (HU) at the region of interest (ROI) of the largest stone, including at least 60% of the stone volume.
  (c) Stone location: lower pole, renal pelvis, ureter, elsewhere, multiple.
(3) Baseline CT metabolic data:
  (a) Visceral fat area (VFA): Measured at the level of the umbilicus, by marking and calculating the fat area internal to the abdominal muscles, found by HU matching fat tissue. We used computer software designed specifically to track these data (Philips Workstation segmentation and tissue measurement tools, Version 12.2.6.2000019, Best, The Netherlands).
  (b) Liver steatosis: Measured in HU for the liver and spleen. To measure the density of the liver parenchyma, we placed an ROI circle in the right lobe of the liver. The circle was sufficiently large to include a large portion of the liver parenchyma while excluding blood vessels. Hepatic attenuation of less than 45 HU is considered in the literature as moderate to severe steatosis [9,10]. The attenuation of the splenic parenchyma was measured in a similar fashion. We also measured the liver–spleen difference (in HU). In the literature, a difference of 19 HU or more is considered as liver steatosis [9].
  (c) Vertebral bone density: We measured ROI attenuation of the body of the L1 vertebra in HU 24.
(4) Baseline blood sample values: Creatinine, uric acid, and calcium, obtained 1 year before or after the baseline NCCT, and at least 4 weeks from the day of NCCT to minimize its effect on these parameters.

### 2.3. Statistical Analysis

Statistical analysis was performed using IBM SPSS Statistics version 25 Armonk, NY, USA, 2020.

Assessment of the correlation between two categorical variables was conducted using either the Pearson Chi-square test or Fisher’s exact test. The strength of linear association between the dependent variable and quantitative continuous variables, with assumed normal distribution, was determined using the *t*-test. For quantitative variables without normal distribution, the non-parametric Mann–Whitney test was used.

Variables that were found on univariate analysis as statistically significant (*p* < 0.05) for being correlated to stone recurrence were entered into a multivariate logistic regression model, to determine which variables could predict stone recurrence. The forward stepwise (likelihood ratio) approach was applied, meaning the variables were entered one after the other, based on their effect on improvement of the model. This method allows maximal neutralization of confounding relationships between variables.

All of the applied tests mentioned above were two-tailed, with significance defined as *p* < 0.05 and confidence interval (CI) of 95%. Survival analysis for categorical variables was performed using the Kaplan–Meier estimator and the log rank test. For continuous variables, Cox regression was used. Receiver operating characteristic (ROC) curves were created to find optimal cutoff values that would predict stone recurrence for continuous variables. 

## 3. Results

We initially reviewed 3600 abdominal CT scans of adults (including CCT and NCCT), of which 350 NCCTs with stones were found. After exclusion of ineligible patients, 237 patients were eventually included in the study analysis. Baseline patient data are presented in Table 1. The mean follow-up period was 6.7 years for patients without recurrence, 4.3 years for patients with recurrence, and 5.8 years overall. Of the 237 patients, 88 (37.1%) had a radiographic recurrence of kidney or ureteral stones according to our definition of recurrence. In subgroup analysis, patients who underwent CT for follow-up imaging (45%) had a recurrence rate of 51%, while patients who had either KUB or US for follow-up imaging (55%) had a recurrence rate of 26%.

On univariate analysis, the following parameters were noted to be significant risk factors for stone recurrence: kidney stone location (kidney vs. ureter) (*p* = 0.007), surgical procedure for removal of stones (*p* < 0.001), baseline calcium (*p* = 0.001), baseline uric acid (*p* = 0.011), and stone burden volume (*p* = 0.044). Other parameters that were not associated with recurrence included age, gender, stone density, and all radiographic-metabolic data (VFA, hepatic attenuation). The full data are shown in Table 2 Table 3 and Table 4.

**Figure 1:**
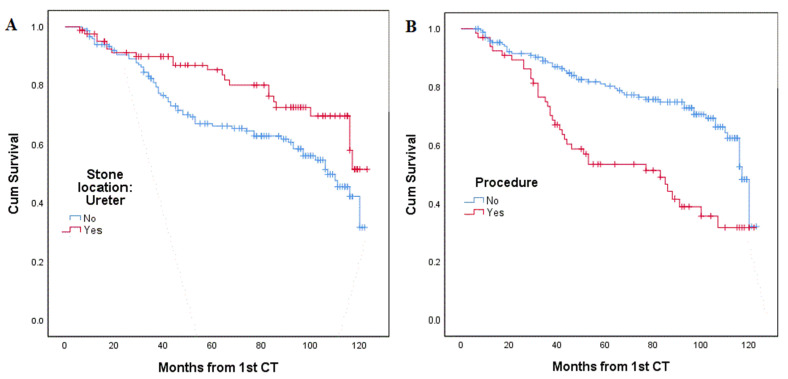
Kaplan–Meier analysis (**A**) stone in the ureter correlated to fewer recurrences, *p* = 0.019; (**B**) undergoing a procedure correlated to higher recurrence rates, *p* < 0.001.

Kaplan–Meier survival analysis showed two categorical variables that were associated with recurrence–stone location and surgical intervention—as presented in Figure 1. Assessment of the initial stone location revealed that 66 patients (43%) of 152 patients with a kidney stone had recurrence, while only 22 patients (26%) of 85 patients with ureteral stone experienced recurrence. Moreover, 37 patients (56%) of 66 patients who underwent surgical intervention had stone recurrence, while only 51 patients (26%) of 171 patients who did not require surgical intervention ultimately had stone recurrence. 

**Figure 2:**
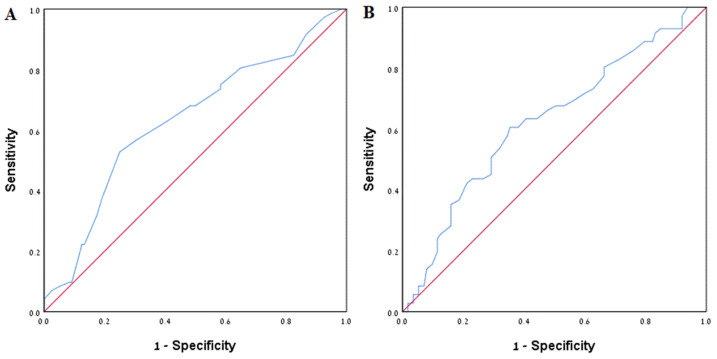
ROC curves. (**A**) ROC curve for baseline calcium; AUC = 0.632; (**B**) ROC curve for baseline uric acid; AUC = 0.624.

On multivariate analysis, the following parameters were associated with recurrence (Table 5): surgical intervention (Odds Ratio (OR) 3.07, *p* = 0.001), higher baseline calcium (OR 2.56, *p* = 0.011), and higher baseline uric acid (OR 1.30, *p* = 0.021), wherein the latter two are separate risk factors. ROC curves for calcium and uric acid are presented in Figure 2. Stone burden volume was not correlated to recurrence (*p* = 0.663), although it was correlated strongly to surgical intervention (*p* < 0.001). 

## 4. Discussion

Nephrolithiasis is widely known as a disease with a tendency to recur. However, the actual recurrence rates and risk factors for recurrence have not yet been adequately established nor have they been studied sufficiently in recent decades. 

To the best of our knowledge, this is one of the largest cohorts in recent decades assessing the natural history of stone disease. While most studies regarding stone recurrence risk factors relied on symptomatic recurrence, we defined stone recurrence based on radiographic findings, which we believe is a more accurate definition. Specifically, we used a strict definition of recurrence: only radiologically-verified new stone formation. This definition avoids the potential inaccuracy of symptomatic recurrence, where a stone that already existed in the initial diagnosis triggers symptoms as it passes. 

Of the 237 included patients, 37% experienced a radiographic recurrence over 4.3 years of follow-up, which corresponds to previously published studies. As expected, the rate of recurrence was higher for patients who had a CT scan for follow-up imaging when compared to those who were followed with either US or KUB. CT is clearly more sensitive than US or KUB for detection of urolithiasis, and it is therefore not surprising that more stones were detected with this modality. The implications of this analysis are that the recurrence rate is probably higher than what was found in this cohort, and that other studies that rely on all imaging modalities rather than only on CT scan results in underestimation of the “true”, higher recurrence rate. Most articles to date focused on symptomatic recurrence and showed slightly lower recurrence rates. Older studies from the 1980s and 1990s noted recurrence rates ranging from 26% to 53% [5,11], while more recent studies demonstrated symptomatic recurrence of 20% and radiographic recurrence of up to 35% [2,12]. It is certainly expected that radiographic recurrence will be higher than symptomatic recurrence because not all recurring stones necessarily present with symptoms. Li et al. [2] reported that the first recurrence, including either symptomatic or radiologic, occurred at a mean of 4.1 years, very similar to our result of 4.3 years. An older, small study based on questionnaires [5] found that the first recurrences occur at 3.5 and 4.6 years for men and women, respectively.

Assessment of epidemiologic, clinical, and radiologic data was done to identify risk factors for stone recurrence. We found that initial identification of stone location in the kidney, as opposed to in the ureter, was correlated significantly to recurrence on both univariate and multivariate analysis. While some studies found no correlation between stone location and recurrence [2,13], or did not test for such correlation [12], a few others do note that stone location appears to be a predictor for stone recurrence. Unal et al. [14] reported that kidney stones were associated with more recurrences compared to ureteric or bladder stones. Moreover, in the ROKS study [3], lower pole and renal calyx stones were associated with high recurrence, while ureterovesical junction stones were associated with fewer recurrences. The higher propensity for recurrence in patients identified with kidney stones may be explained by possibly different mechanisms of stone formation. While kidney stone formation may reflect a combination of metabolic risk factors such as urine acidity, supersaturation and genetic tendency, small ureterolithiasis may be the result of a single acute event such as severe dehydration. In this context, patients with kidney stones carry risk factors chronically, and are thus more prone for recurrences. Another possible explanation is that patients with ureteral stones may resolve spontaneously, before any imaging takes place, and will therefore be underdiagnosed. 

Our results also demonstrated a correlation between endourological procedure (extracorporeal shockwave lithotripsy, endoscopy, or percutaneous nephrolithotomy) and recurrence with OR = 3.07. Moreover, stone burden was correlated strongly to procedure (*p* < 0.001), which supports our hypothesis of confounding factors associated with higher stone burden. In the ROKS study [3] and a revision study [15], surgical intervention was weakly associated with fewer symptomatic recurrences. Recent literature lacks an attempt to assess the initial procedure on the first event as a predictor for recurrence [12,13,16], but a few studies use surgery as an outcome that marks recurrence [13,17]. Our results demonstrate that surgery is a marker of more severe stone disease, therefore accounting for higher recurrence rates. Overall, results in our study and in the literature regarding surgical intervention and stone burden are mixed, and may present confounding factors; for instance, patients with a high burden may be subject to closer surveillance and more surgical treatments. 

Higher baseline calcium (OR = 2.56), as well as higher uric acid (OR = 1.30), were also found as *independent* predictors for stone recurrence. Hypercalcemia and hypercalciuria are known risk factors for nephrolithiasis [1,18]. Hyperuricemia is also an established risk factor for stone disease [1] and has been related to MetS, another independent risk factor [1,19,20]. Interestingly, our results imply that a higher level of calcium and/or uric acid is linked to a higher risk of recurrence, even if levels remain within the range of normal values. Similar results were observed by Unal et al. [14], in which calcium levels above 9.2 mg/dL, still within normal values, were significantly associated with stone recurrence.

To identify an optimal cutoff value of baseline calcium and baseline uric acid that would better predict recurrence, we considered ROC curves (Figure 2). The optimal cutoff values, determined by maximum sum of sensitivity and specificity, were 9.45 mg/dL calcium with Area under the ROC Curve (AUC) of 0.632 (sensitivity 0.528, specificity 0.75) and 5.65 mg/dL uric acid and AUC of 0.624 (sensitivity 0.606, specificity 0.646). We have no clear explanation as to why higher levels of calcium and/or uric acid even within normal limits impact stone recurrence. However, a similar situation may be learned from the LDL cholesterol control guidelines, where target values are stratified according to risk factors to develop cardiovascular disease. 

Gender and age were not correlated to recurrence. Insignificance of gender is consistent with our hypothesis and is supported by the literature; as in most recent studies, gender has not been found to be correlated to recurrence [2,12,13,14]. The mean patient age in our study was 53 years, corresponding to the known literature. The influence of age on recurrence is still not clear. In some studies, younger age was found to be a predictor for recurrence [3,13], while, in others, as in our study, it was not [2,12,14]. 

None of the radiographic-metabolic variables were correlated with stone recurrence. These variables include VFA representing obesity, splenic and hepatic attenuation representing hepatic steatosis and reliable markers of MetS [9,10,21,22], and vertebral bone density, representing osteoporosis and linked to urolithiasis [23,24,25]. Obesity and high body mass index (BMI), markers of the waist circumference component of MetS, are known risk factors for stone disease [20,26]. However, a direct correlation between BMI and recurrence of stones has rarely been found; for example, Lee et al. [20] reported higher BMI being associated with recurrence only in men. In recent studies regarding stone recurrence, specific components of MetS, such as high triglycerides and obesity, were not associated with recurrence [2,3]. One study associated diabetes with stone growth [2], while insulin resistance has been linked to stone formation [26]. In one large study [19], high blood pressure was directly associated with stone disease, but not other specific criteria of MetS. Although there is consistent evidence of the correlation between MetS and occurrence of stone disease, MetS has not yet been correlated directly to recurrence of stones, which is perplexing.

The study has several limitations. Our cohort included patients that underwent NCCT at our hospital for any reason, so that we are prone to selection bias. Another limitation is information bias due to our ability to collect information only from the hospital data system; patients who underwent surveillance in other medical systems may have had imaging reports we could not access. To minimize this bias, a large sample size was examined. Moreover, in contrast to other retrospective series, we believe that most of our patients continued with follow-up imaging studies at the same hospital, which allowed us to closely monitor our patients over a long period of time. It is possible, of course, that some of the patients passed stones prior to any imaging examination. These patients may have experienced symptomatic recurrence that was not tracked, as no imaging would have been done. It is very likely, though, that symptomatic recurrence would be lower than radiographic recurrence, so that, ultimately, this would have only a minimal effect on our findings. In addition, we lack information as to whether the patients included in this study had prior kidney stones events, which potentially could have influenced the time to recurrence. Finally, we had scarce information, at best, regarding whether or not patients had co-morbidities or were treated medically by either lifestyle changes or medications; such treatments would have an impact on the course of the disease. However, it is a reasonable assumption that most symptomatic patients had lifestyle counselling from their medical caregivers. 

## 5. Conclusions

In this retrospective cohort study, the effect of multiple demographic, metabolic and radiographic characteristics on radiologic kidney stone recurrence was evaluated. We found that 37% of the patients will experience recurrence after a mean of 4.3 years. Higher baseline calcium and/or uric acid (independently), initial identification of stone location in the kidney, and surgical intervention were significant risk factors for stone recurrence. These findings may assist in tailoring patient surveillance protocols based on characteristics upon diagnosis. Applying personal follow-up plans may reduce unnecessary physician visits and redundant imaging tests, which increase health costs and loss of workdays, as well as increased radiation exposure in patients who are less likely to have early recurrence. In addition, these findings may aid in the early diagnosis and treatment of high-risk patients and decrease the rate of symptomatic recurrence. 

## Figures and Tables

**Table 1:** Baseline demographic, clinical and imaging characteristics of patients (*n* = number; SD = standard deviation; HU = Hounsfield units; VFA = visceral fat area).

Characteristic	Value
Gender	
Male, *n* (%)	167 (70.5%)
Female, *n* (%)	70 (29.5%)
Age, years, mean (SD)	53.15 (15.58)
Stone location	
Ureter, *n* (%)	85 (35.9%)
Lower pole, *n* (%)	21 (8.9%)
Renal pelvis, *n* (%)	9 (3.8%)
Elsewhere, *n* (%)	27 (11.4%)
Multiple, *n* (%)	95 (40.1%)
Underwent stone-related procedure, *n* (%)	66 (27.8%)
2nd imaging modality	
NCCT, *n* (%)	85 (35.9%)
CCT, *n* (%)	21 (8.9%)
US, *n* (%)	124 (52.3%)
KUB, *n* (%)	7 (3.0%)
Baseline creatinine, mg/dL, mean (SD)^†^	0.96 (0.27)
Baseline calcium, mg/dL, mean (SD)^†^	9.27 (0.48)
Baseline uric acid, mg/dL, mean (SD)^†^	5.52 (1.46)
Stone burden volume, mm^3^, mean (SD)	513.5 (1720.60)
Stone density at ROI, HU, mean (SD)	723.12 (307.05)
VFA, cm^2^, mean (SD) ^†^	172.99 (69.27)
Hepatic attenuation, HU, mean (SD	48.62 (13.31)
Splenic attenuation, HU, mean (SD)^†^	42.37 (7.06)
Liver attenuation index, HU, mean (SD)^†^	6.25 (11.84)
Bone density at L1, HU, mean (SD)	149.60 (51.64

^†^ We could not retrieve these data from all patients.

**Table 2:** Association between categorical variables and stone recurrence, univariate analysis. Analysis in this table was conducted using Fisher’s exact test (*n* = number). Boldface indicates statistically significant factors.

Variable	*Recurrence*		*p*-Value
	*No, n (%)*	*Yes, n (%)*	
Gender			
Male, *n* (%)	104 (62.3%)	63 (37.7%)	*0.77*
Female, *n* (%)	45 (64.3%)	25 (35.7%)	
Stone Location			
Kidney, *n* (%)	86 (56.6%)	66 (43%)	***0.007***
Ureter, *n* (%)	63 (74.1%)	22 (25.9%)	
Underwent stone-related procedure			
No, *n* (%)	120 (70.2%)	51 (29.8%)	***<0.001***
Yes, *n* (%)	29 (43.9%)	37 (56.1%)	

**Table 3:** Association between continuous numerical variables and stone recurrence, univariate analysis (*n* = number). Boldface indicates statistically significant factors.

**Normal Distribution Assumed, *t*-Test, 2-Tailed**			
**Variable**	***Recurrence***		***p*** **-Value**
	***No, Mean***	***Yes, Mean***	
Age at 1st CT	54.17 years	51.43 years	*0.192*
VFA	171.76 cm^2^	175.10 cm^2^	*0.721*
Hepatic attenuation	49.09 HU	49.09 HU	*0.481*
Splenic attenuation	42.54 HU	42.08 HU	*0.626*
Liver attenuation index	6.55 HU	5.75 HU	*0.617*
Bone density at L1	147.13 HU	153.78 HU	*0.339*
Baseline creatinine	0.95 mg/dL	0.98 mg/dL	*0.550*
Baseline calcium	9.19 mg/dL	9.42 mg/dL	***0.001***
Baseline uric acid	5.31 mg/dL	5.86 mg/dL	***0.011***
**Non-normal distribution, Mann-Whitney test, 2-tailed**			
**Variable**			***p*** **-Value**
Stone burden volume			***0.044***
Stone density at ROI			*0.254*

**Table 4:** Univariate Cox regression survival analysis for continuous variable. Boldface indicates statistically significant factors.

Variable	*p*-Value	Hazard Ratio (CI 95%)
Age at 1st CT	*0.166*	
VFA	*0.584*	
Hepatic attenuation	*0.269*	
Splenic attenuation	*0.643*	
Liver attenuation index	*0.141*	
Baseline creatinine	*0.530*	
Baseline calcium	***0.002***	1.935 (1.27–2.96)
Baseline uric acid	***0.035***	**1.174 (1.012–1.362)**
Stone burden volume	*0.661*	
Stone density at ROI	*0.228*	

**Table 5:** Multivariate analysis, logistic regression. Boldface indicates statistically significant factors.

Variable	*p*-Value	OR (CI 95%)
Underwent stone-related procedure	***0.001***	3.071 (1.560–6.046)
Baseline calcium	***0.011***	2.564 (1.243–5.290)
Baseline uric acid	***0.021***	1.303 (1.040–1.633)
Stone burden volume	*0.663*	---
Stone location in kidney ^†^	***0.012***	2.163 (1.181–3.962)

^†^ Performed with calcium and uric acid excluded.

## Data Availability

The data presented in this study are available on request from the corresponding author. The data are not publicly available due to health fund and hospital policy regarding patient confidentiality.
